# Safety and efficacy of prophylaxis for *Pneumocystis jirovecii* pneumonia involving trimethoprim-sulfamethoxazole dose reduction in kidney transplantation

**DOI:** 10.1186/s12879-019-3944-0

**Published:** 2019-04-05

**Authors:** G. V. Ramesh Prasad, Jill Beckley, Mohit Mathur, Madhushankar Gunasekaran, Michelle M. Nash, Lindita Rapi, Michael Huang, Jeffrey S. Zaltzman

**Affiliations:** 1 Kidney Transplant Program, St. Michael’s Hospital, University of Toronto, 61 Queen Street East, 9th Floor, Toronto, ON M5C 2T2 Canada; 2 Department of Medicine, University of Toronto, Toronto, ON Canada

**Keywords:** Adverse effects, Hyperkalemia, Leukopenia

## Abstract

**Background:**

Trimethoprim-sulfamethoxazole (TMP-SMX) is the drug of choice for anti-*Pneumocystis jirovecii* pneumonia (PcP) prophylaxis in kidney transplant recipients (KTR). Post-transplant management balances preventing PcP with managing TMP-SMX-related adverse effects. TMP-SMX dose reduction addresses adverse effects but its implications to incident PcP are unclear.

**Methods:**

We performed a retrospective review of all patients transplanted between 2011 and 2015 prescribed daily single strength TMP-SMX for twelve months post-transplantation as PcP prophylaxis. Actual TMP-SMX dose and duration, adverse effects, number of dose reductions and reasons, and PcP events were captured. Multivariate logistic regression analyses for risk factors associated with dose reduction were performed.

**Results:**

Of 438 KTR, 233 (53%) maintained daily TMP-SMX and 205 (47%) sustained ≥1 dose reduction, with the point prevalence of a reduced dose regimen being between 18 and 25%. Median duration for daily TMP-SMX was 8.45/12 months, contributing 4137 patient-months daily TMP-SMX and 1110 patient-months with a reduced dose. PcP did not occur in any patients. There were 84 documented dose reductions for hyperkalemia and 102 for leukopenia, with 12 and 7 patients requiring TMP-SMX cessation. In multivariate analysis, a living donor transplant protected against hyperkalemia (Odds Ratio 0.46, 95% CI 0.26–0.83, *p* < 0.01) while acute rejection risked leukopenia (Odds Ratio 3.31, 95% CI 1.39–7.90, *p* = 0.006).

**Conclusions:**

TMP-SMX dose reduction is frequent in the first post-transplant year but PcP does not occur. To limit the need for TMP-SMX dose reduction due to adverse effects, a clinical trial comparing daily to thrice weekly single strength TMP-SMX in de-novo KTR is justified.

## Background

*Pneumocystis jirovecii* (*P. jirovecii*) is an important pneumonia-causing opportunistic fungus in kidney transplant recipients (KTR), causing disease (*Pneumocystis jirovecii* pneumonia, or PcP) through reactivation, as well as direct or indirect person-to-person transmission [1]. PcP associates with a high mortality rate in solid organ transplant recipients [2, 3]. The drug of choice for anti-Pneumocystis prophylaxis is trimethoprim-sulfamethoxazole (TMP-SMX) [1]. However, TMP-SMX associates with numerous adverse effects, including bone marrow suppression, hepatitis, hyperkalemia, rash, interstitial nephritis, aseptic meningitis, and pancreatitis [1]. Post-transplant management therefore becomes a balancing act between preventing PcP on the one hand and managing the adverse effects of TMP-SMX on the other.

All other PcP prophylactic agents are considered second choice to TMP-SMX because of their relatively unfavorable breadth of coverage, tolerability, cost, and efficacy [1]. Even with all of its known adverse effects, TMP-SMX will remain a mainstay of PcP prevention for the foreseeable future. Despite consensus about the role of TMP-SMX in routine post-transplant care, there remains a remarkable vagueness about how TMP-SMX should best be used for PcP prophylaxis. The current recommendation for TMP-SMX use ranges from single strength (80 mg TMP/400 mg SMX) to double strength (160 mg TMP/800 mg SMP) orally, either daily or three times weekly, for at least 6 to 12 months [1], with little evidence available to clinicians to prefer one dosing regimen over another. The 5th European Conference on Infections in Leukemia (ECIL-5) guidelines suggest that TMP-SMX given two or three times weekly is the drug of choice for PcP primary prophylaxis in stem cell transplant recipients [4]. As a result, kidney transplant programs are free to choose their own TMP-SMX-containing PcP prophylactic regimens within these wide boundaries. The choice of prophylactic regimen is not trivial. Prescribing the best tolerated and efficacious regimen using TMP-SMX through a narrower recommendation about TMP-SMX use will benefit transplant recipients and programs both by optimizing monitoring protocols and reducing intervention efforts related to drug toxicity, and at the same time prevent PcP.

There are no prospective randomized controlled trials (RCT) related to optimizing TMP-SMX use in KTR. As one step toward designing a clinical trial to determine the ideal TMP-SMX dose and duration for PcP prophylaxis, we retrospectively examined the efficacy and safety of daily single strength TMP-SMX prescribed as PcP prophylaxis for twelve months post-kidney transplantation. Our hypothesis was that thrice weekly single strength TMP-SMX is at least as effective as daily TMP-SMX for PcP prophylaxis in KTR.

## Methods

St. Michael’s Hospital (SMH) in Toronto, Canada is a university-affiliated tertiary care medical-surgical centre that currently performs 120–150 adult single-organ kidney transplant procedures annually and provides long-term follow-up to approximately 1700 KTR. We performed a retrospective review of the electronic records of all patients transplanted at SMH between January 1, 2011 and June 30, 2015, who were prescribed daily single strength TMP-SMX during their initial hospital admission with the intent to continue TMP-SMX for twelve months post-transplantation as PcP prophylaxis. If TMP-SMX dose reduction is deemed necessary, the frequency of TMP-SMX administration is typically reduced to thrice weekly, and then stopped entirely if further reduction is deemed necessary. Since 2009 all KTR receive two induction doses of perioperative basiliximab, once-daily tacrolimus (Advagraf®), mycophenolate mofetil or mycophenolic acid, and prednisone as part of their initial immunosuppressive medication regimen. Anti-thymocyte globulin is used sparingly, even in recipients deemed to be at high immunological risk. Post-transplant laboratory monitoring in the first post-transplant year is performed twice weekly for the first three months, weekly for the next three months, and then once every two weeks to the end of 9 months and monthly thereafter.

Patients were excluded from this analysis if they received a multi-organ transplant, were transplanted at another centre, or were initially prescribed a different strength of TMP-SMX or another drug for PcP prophylaxis. Patient demographics as well as the occurrence of post-transplant events such as delayed graft function (defined as the need for dialysis in the first post-transplant week), acute rejection (based on Banff criteria), cytomegalovirus IgG antibodies and new-onset diabetes, were collected by chart review. The dose and duration of TMP-SMX prophylaxis, other medications prescribed during the first year, adverse effects attributed to TMP-SMX as documented by the clinician, number of dose reductions of TMP-SMX and the corresponding reasons including adverse effects attributed to TMP-SMX, and PcP events were captured from the electronic medical record. All data were verified by at least two investigators.

The primary outcome was the incidence of PcP per 100 patient-months during periods of daily TMP-SMX or reduced-dose TMP-SMX. Secondary outcomes included adverse effects attributable to TMP-SMX that necessitated dose reduction including hyperkalemia, defined as serum potassium ≥5.5 mmol/L; total leukopenia, defined as total WBC ≤ 3000/cu mm; absolute neutropenia, defined as neutrophil count ≤500/cu mm; thrombocytopenia, defined as platelet count ≤100,000/cu mm; anemia, defined as hemoglobin ≤100 g/L. and hepatitis, defined as alkaline phosphatase ≥125 IU/mL, alanine aminotransferase ≥45 IU/mL, and/or aspartate aminotransferase ≥40 IU/mL. The number and timing of dose reductions or alterations in TMP-SMX over the first year post-transplant were recorded. Since the dose per se of TMP-SMX was always single strength, dose reduction was equivalent functionally to a reduction in dose frequency. All patterns of dose reduction (such as gradual reduction or complete discontinuation) were combined into a single outcome variable.

Comparisons were made by chi-square or Fisher exact testing for categorical variables and by paired or unpaired student t-test or analysis of variance for continuous variables as appropriate, followed by multivariate logistic regression analysis. Since patients may have been switched back-and-forth between the full-dose and reduced-dose regimes during the first year post-transplant, patients contributed follow-up time to both groups. A *p*-value< 0.05 was considered significant for all comparisons. SAS version 9.2 (Cary, NC, USA) was the statistical software used for all analyses. The study was approved by the Research Ethics Board at St. Michael’s Hospital, Toronto, protocol number REB 16–210.

## Results

**Fig. 1:**
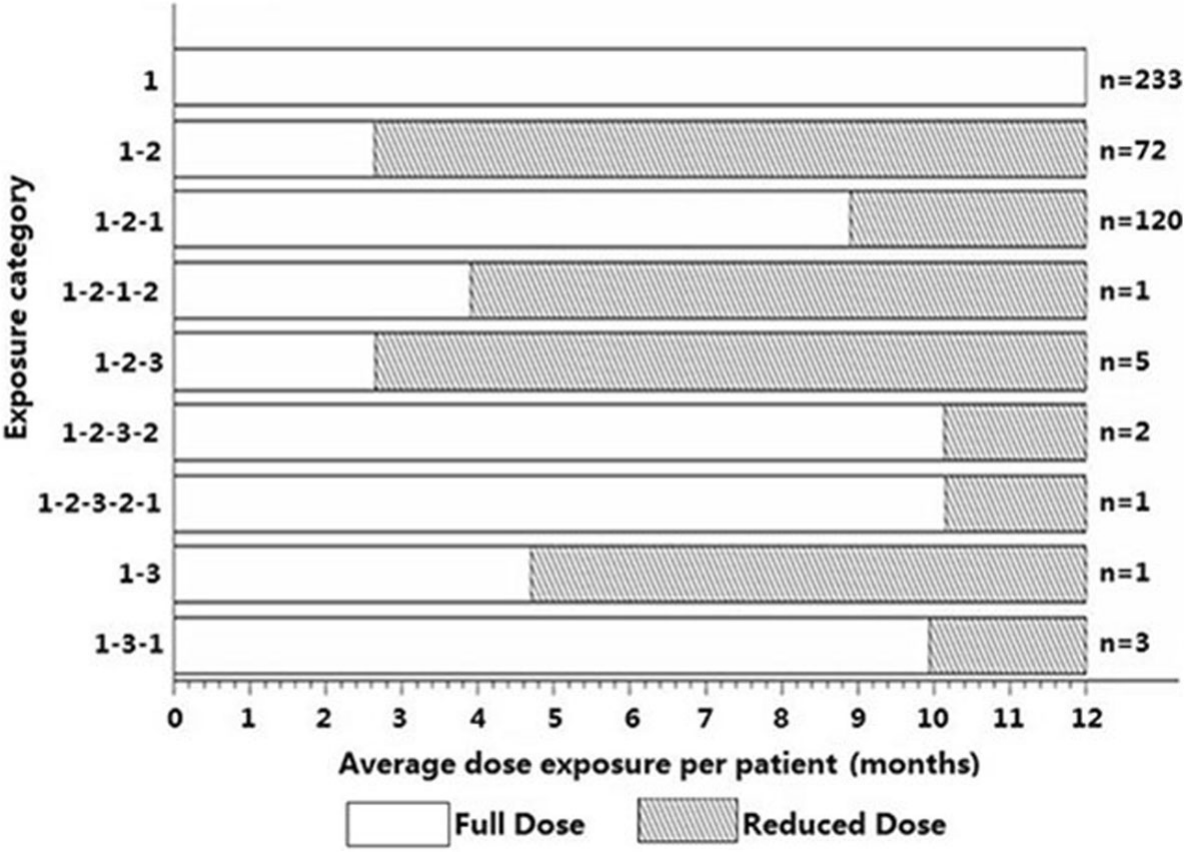
Patterns of trimethoprim-sulfamethoxazole (TMP-SMX) dose reduction in kidney transplant recipients (*N* = 438). “Full dose” refers to daily single strength TMP-SMX while “reduced dose” refers to any dosing less than once daily. 1 = daily, 2 = thrice weekly, 3 = twice weekly or none

There were 438 KTR who met entry criteria for the study, after the exclusion of 33 patients receiving either dapsone or atovaquone at any time in the first 12 months post-transplantation. Of 438 KTR, 233 (53%) maintained a full dose of TMP-SMX for 12 months and 205 (47%) sustained at least one dose reduction of TMP-SMX over the 12-month follow-up period (Fig. 1). A comparison of demographic and post-transplant characteristics between these two groups of patients is provided in Table 1. There were only 12 patients whose TMP-SMX dose was reduced to less than thrice weekly, precluding a separate analysis of that group (Fig. 1). Out of 438 patients, 112 were receiving a reduced dose of TMP-SMX at 3 months, 105 at 6 months, 88 at 9 months, and 80 at 12 months. Therefore, while the overall incidence of TMP-SMX dose reduction was 47%, at any given time the prevalence of a reduced dose was 18–25%. The median duration for full dose TMP-SMX was 8.45 months and the median duration for reduced dose TMP-SMX was 5.07 months. The risk factors associated with TMP-SMX dose reduction were receipt of a deceased donor organ, history of hemodialysis prior to transplantation, and acute rejection (Table 1). Allograft function was reduced in the reduced dose group at 3 months (serum creatinine 124 ± 45 v 113 ± 36 μmol/L, *p* = 0.009) and 6 months (serum creatinine 122 ± 45 v 110 ± 33 μmol/L, *p* = 0.05) but this difference had resolved at 12 months (serum creatinine 129 ± 89 v 119 ± 14 μmol/L, *p* = 0.40). There were no cases of PcP in either group and there were no patient hospitalizations or deaths directly attributed to TMP-SMX-related adverse effects. There were also no documented cases of infection with Nocardia, Listeria, or Toxoplasma in either group.

**Table 1:** Comparison of demographic and post-transplant characteristics between patients maintaining a full dose and patients with at least one dose reduction in trimethoprim-sulfamethoxazole in the first 12 months post-kidney transplantation

Parameter	Full Dose Group (*N* = 233)	Reduced Dose Group (*N* = 205)	*P* value
Age at transplantation (years) (mean ± SD, range)	52.5 ± 13 (19–81)	54.1 ± 12 (24–76)	0.22
Sex (*N*, %)			0.42
Male	144 (62)	119 (58)	
Female	89 (38)	86 (42)	
Donor Source (*N*, %)			0.002
Deceased	142 (61)	154 (75)	
Live	91 (39)	51 (25)	
Number of transplants			0.4
One	224 (97)	194 (96)	
Two	7 (3)	9 (4)	
Cause of end-stage kidney disease (*N*, %)			
Diabetes	41 (18)	44 (22)	0.30
Hypertension	25 (11)	19 (9)	0.61
Glomerulonephritis	94 (40)	78 (38)	0.62
Polycystic kidney disease	23 (10)	28 (14)	0.21
Congenital kidney disease	10 (4)	7 (3)	0.63
Others	40 (17)	29 (14)	0.28
Ethnicity			
Caucasian	94 (40)	70 (34)	0.13
Black	16 (7)	21 (10)	0.20
East Asian	32 (14)	20 (10)	0.19
South Asian	47 (20)	53 (26)	0.15
Other	43 (19)	41 (20)	0.71
Pre-transplant dialysis modality (*N*, %)			0.015
Hemodialysis	122 (53)	131 (64)	
Peritoneal dialysis	69 (30)	54 (27)	
Preemptive	36 (17)	18 (9)	
Dialysis duration (days) (mean ± SD)	1790 ± 1315	2052 ± 1427	0.06
Cytomegalovirus IgG antibody *N* (%)	174 (75)	167 (81)	0.18
Epstein Barr virus IgG antibody *N* (%)	211 (91)	189 (93)	0.70
Hepatitis B surface antigen *N* (%)	4 (2)	3 (1)	0.29
Hepatitis C antibody *N* (%)	4 (2)	2 (1)	0.41
HIV antibody *N* (%)	2 (1)	4 (2)	0.29
Tuberculin skin test positive *N* (%)	31 (13)	11 (5)	0.33
Cumulative Panel Reactive Antibody (%)(mean ± SD, range)	7.5 ± 20 (0–99)	13.5 ± 28 (0–100)	0.02
History of smoking (*N*, %)	94 (42)	56 (28)	0.003
Delayed graft function *N* (%)	38 (16)	41 (20)	0.31
Acute rejection *N* (%)	7 (3)	18 (9)	0.009
Post-transplant diabetes (*N*, %)	19 (8)	16 (8)	0.81
Graft loss *N* (%)	2 (1)	0 (0)	0.28
Immunosuppressive medication at discharge (*N*, %)			
Tacrolimus	215 (93)	189 (93)	0.99
Cyclosporine	11 (5)	14 (7)	0.34
Mycophenolate mofetil	104 (45)	80 (39)	0.23
or mycophenolic acid	103 (45)	95 (44)	0.64
Azathioprine	11 (5)	4 (2)	0.09
Prednisone	219 (95)	196 (97)	0.37
Other medication (*N*, %)			
Angiotensin-converting enzyme inhibitor	12 (5)	9 (4)	0.71
Angiotensin II receptor blocker	19 (8)	24 (12)	0.21
Non-dihydropyridine calcium channel blocker	21 (9)	13 (6)	0.28
Dihydropyridine calcium channel blocker	100 (43)	100 (49)	0.21
Beta blocker	87 (37)	90 (44)	0.15
Alpha blocker	23 (10)	19 (9)	0.83
Loop diuretic	10 (4)	12 (6)	0.29
Thiazide diuretic	4 (2)	4 (2)	0.56

Abbreviations: IgG immunoglobulin G, SD standard deviation

There were a total of 4137 patient-months of follow-up with a full dose across both the full dose and reduced groups, of which 3257 patient-months occurred prior to any dose reduction and 879 months after the reversal of any dose reduction. Similarly, there were 1110 patient-months of follow-up with a reduced dose. The dose reduction followed several patterns, as illustrated in Fig. 1. The median time-to-dose reduction based on indication was 16 days for thrombocytopenia, 136 days for leukopenia, 173 days for hyperkalemia, 228 days for anemia, and 322 days for hepatitis, despite the temporal prevalence of these conditions at varying times throughout the 12 month period for the two TMP-SMX doses. An estimated glomerular filtration rate (eGFR) < 30 ml/min/1.73m^2^ was present in 45 patients overall.

There were 84 documented dose reductions for hyperkalemia, corresponding to an overall risk for dose reduction of 0.39/100 patient-months. Risk for dose reduction was highest in the first 3 months post-transplantation at 0.54/100 patient-months. Twelve patients required a further TMP-SMX dose reduction due to persistent hyperkalemia. A living donor transplant was protective against hyperkalemia in multivariate analysis (Odds Ratio 0.46, 95% CI 0.26–0.83, *p* = 0.01); all other conventional risk factors for hyperkalemia were insignificant. However, there was a strong interaction between hyperkalemia and leukopenia. Patients with leukopenia were more likely to develop hyperkalemia as well (Odds Ratio 5.14, 95% CI 1.67–15.78, *p* = 0.004).

There were 102 documented dose reductions for leukopenia, corresponding to an overall risk for dose reduction of 2.32/100 patient-months. The risk for dose reduction was again highest in the first 3 months, at 4.05/100 patient-months. Seven patients required a further TMP-SMX dose reduction due to persistent leukopenia. Only acute rejection was independently associated with leukopenia (Odds Ratio 3.31, 95% CI 1.39–7.90, *p* = 0.006). Modifying age (e.g. to greater than 50 years) did not affect the multivariate result analysis.

Other reasons for TMP-SMX dose reduction included thrombocytopenia and/or anemia in 5 patients and hepatitis in 7 patients. These smaller numbers of these events were not analyzed further. There were no documented cases of TMP-SMX dose reduction or discontinuation due to skin rash or allergic interstitial nephritis.

## Discussion

In this retrospective single-centre cohort analysis of contemporary KTR, both the daily and reduced-frequency administration of single strength TMP-SMX effectively prevented PcP in the first post-transplant year. However, a protocol of initiating daily single strength TMP-SMX associates with significant adverse effects. Almost one-half of KTR required a TMP-SMX dose reduction, mostly due to hyperkalemia and leukopenia. The only discernible risk factor for hyperkalemia was donor source while that for leukopenia was acute rejection; those at risk for one were more likely to be at risk for the other. Such KTR were at additional risk for requiring further dose reductions, but fortunately there was no hospitalization or mortality directly attributable to TMP-SMX. Nonetheless, the efforts required to monitor for TMP-SMX-related adverse effects to prevent such serious outcomes mandates efforts toward evaluating TMP-SMX prophylactic regimens in KTR with less dose exposure than once daily single strength TMP-SMX, while still effectively preventing PcP.

Pneumocystis infection was first described in the lungs of rats in 1909 and in humans in 1942 [5]. The risk of infection is highest between two and six months post-transplant, reaching 14% without prophylaxis [4]. Exposure to the organism is ubiquitous [6]. PcP can occur through both reactivation and acquisition of a new strain by person-to-person transmission, and infection by more than one strain is possible [6]. Immunodeficiency or immunosuppression is a prerequisite to infection, However, unlike in HIV-infected patients, in transplant recipients an acute respiratory illness is much more common [7]. TMP-SMX was introduced in 1968 [8]. TMP-SMX reduces the risk for non-HIV related PcP by over 90% [9]. A large randomized trial in over 2500 HIV-infected patients demonstrated that TMP-SMX effectively prevents PcP when administered as a double-strength tablet daily or thrice weekly [10]. TMP-SMX was discontinued in up to 20% in the daily and 10% of the thrice weekly groups, after which PcP occurred in many instances. These data cannot readily be extrapolated to transplant recipients [11], although a systematic review indicates safety and efficacy of daily single-strength TMP-SMX and alternate-day double-strength TMP-SMX [12]. There are no similar clinical trial data involving alternate-day or thrice weekly single-strength TMP-SMX in both immunocompromised and immunosuppressed populations. In the present study TMP-SMX dosing was typically first reduced in almost 50% of patients, and then discontinued only as a last resort in a small minority. Since no PcP occurred, a clinical trial of daily versus thrice weekly single-strength TMP-SMX at preventing PcP in KTR while avoiding drug dosing changes due to adverse effects seems justified due to the very high dose reduction rates associated with daily single-strength TMP-SMX. Such a trial would be difficult to power for PcP events since they are uncommon, but could be powered to detect clinically important reductions in specific adverse effects.

TMP causes hyperkalemia by blocking apical membrane sodium channels in the mammalian distal nephron to reduce transepithelial voltage and potassium secretion [13]. Although hyperkalemia is reversible, it may be life-threatening [14]. Risk factors include older age, diabetes, renal insufficiency, and drugs such as angiotensin-converting enzyme inhibitors (ACE-I) and angiotensin receptor blockers ARB), among others [8]. Renal insufficiency and ACE-I or ARB use together may cause hyperkalemia even with TMP-SMX doses lower than single-strength daily [15]. Conversely, beta-blockers do not seem to confer added risk [16]. Tacrolimus also leads to hyperkalemia in KTR [17]. In the present study neither ACE-I/ARB use nor beta blocker use was associated with a dose reduction in TMP-SMX for hyperkalemia, perhaps because these drugs are stopped as a first measure prior to TMP-SMX dose reduction. Similarly, the significance of concomitant leukopenia may indicate that patients requiring TMP-SMX dose reduction are especially susceptible to developing multiple drug-related adverse effects.

TMP-SMX also associates with leukopenia that can be prolonged [18]. Leukopenia is typically neutropenia, either through impaired granulopoiesis from tetrahydrofolate synthesis inhibition or though peripheral destruction as part of an idiosyncratic reaction [19]. Lymphocyte counts are typically preserved even when patients are leukopenic. Incident hematologic adverse effects from TMP-SMX, as a whole, rank second to hypersensitivity reactions in the HIV population [10]. In the present study, dose reduction for leukopenia was very common, unlike hypersensitivity reactions that were rarely documented. Risk factors for TMP-SMX associated leukopenia may include concomitant mycophenolate or azathioprine therapy, although this may be confounded by corticosteroid therapy, and viral infections.

Due to the retrospective nature of the study and the fact that all patients were initially started on daily TMP-SMX, however, we cannot exclude the possibility that the beneficial effects of preventing PcP by daily oral TMP-SMX administration persisted for a length of time after conversion to a reduced frequency regime. This study provides justification for proceeding with a randomized trial in de-novo KTR to evaluate daily versus reduced frequency oral single-strength TMP-SMX to prevent PcP in the first year after kidney transplantation, after consideration of immunological risk and the consequent need for enhanced immunosuppression, as well as the possibility of a hybrid strategy requiring different dosing regimens in the early and late part of the first post-transplant year. The present study is also limited by the lack of information about drug non-adherence related to TMP-SMX, or other possible benefits of TMP-SMX prophylaxis including the occurrence of bacterial urinary tract infections. Pneumocystis colonization can also be a challenging problem in transplant recipients despite the use of TMP-SMX prophylaxis, but we were unable to ascertain potentially valuable information concerning asymptomatic infection in this population. Based on the current study, however, reduced TMP-SMX dosing may be efficient enough to prevent PcP after solid organ transplantation. There were also very few patients whose TMP-SMX dose was reduced to less than thrice weekly to allow for an efficacy comparison to a twice weekly or full dose TMP-SMX regimen.

Dose reductions of TMP-SMX require a considerable amount of staff time, leading to increased overall costs for transplant programs, and the adverse effects that necessitated dose reduction in the first place will entail potentially increased patient morbidity and mortality unless they are promptly addressed. Starting with a reduced dose of TMP-SMX, such as single-strength TMP-SMX thrice weekly, carries advantages to both clinicians and patients provided PcP can be prevented at the same time. The transplant community has established with reasonable certainty that daily double-strength TMP-SMX is not necessary; what still requires further investigation is whether even daily single-strength TMP-SMX is needed. If thrice weekly single-strength TMP-SMX for a year is proven sufficient for preventing PcP, then extending such therapy beyond the first year post-transplant, when patients are seen less frequently, can be tested as a strategy to minimize the occurrence of late-onset PcP as well.

## Conclusions

In conclusion, TMP-SMX dose reduction is frequent in the first post-transplant year, but PcP does not occur after dose reduction at least during the rest of the first post-transplant year. To confirm that we can limit the need for such frequent TMP-SMX dose reduction resulting from adverse effects, a clinical trial in KTR comparing daily single strength to thrice weekly single strength TMP-SMX in de-novo KTR is justified.

## Abbreviations

ACE-I: Angiotensin converting-enzyme inhibitor
ARB: Angiotensin II receptor blocker
CI: Confidence interval
eGFR: estimated glomerular filtration rate
IgG: Immunoglobulin G
KTR: Kidney transplant recipients
PcP: *Pneumocystis jirovecii* pneumonia
SD: Standard deviation
SMH: St. Michael’s Hospital
TMP-SMX: Trimethoprim-sulfamethoxazole
